# Comment on “Methotrexate-Induced Accelerated Nodulosis: A Case Series”

**DOI:** 10.31138/mjr.171125.voc

**Published:** 2026-03-01

**Authors:** Pavlos Stamatis

**Affiliations:** 1 Rheumatology, Department of Clinical Sciences, Lund University, Lund, Sweden;; 2 Rheumatology, Sunderby Hospital, Luleå, Sweden

**Keywords:** rheumatoid arthritis, methotrexate, subcutaneous nodules, methotrexate-induced accelerated nodulosis

## Abstract

Comment on “Methotrexate-Induced Accelerated Nodulosis: A Case Series” [Subramanian R et al, Mediterr J Rheumatol 2024;35(4):680–683].

I read with great interest the article by Sabmaranian et al. on methotrexate-induced accelerated nodulosis (MIAN), presented in the context of five clinical cases.^1^ In the introduction, the authors describe MIAN as an uncommon manifestation. However, in the discussion, they refer to an estimated incidence ranging from 8% to 11.6%. Although the authors do not directly cite the source, by looking at the reference list, I suspect that these frequency estimates were drawn from the following three articles; by Kremer et al., Kerstens et al., and Patatanian et al.^1–4^ In my opinion, this figure is inflated and misrepresets methotrexate’s safety profile. I would therefore like to present the following arguments.

One of the early studies cited is by Kremer and Lee, investigating the long-term safety of methotrexate in 29 patients with high disease activity who had failed prior therapies available at the time.^2^ In this study, 21/29 patients were RF-positive, and at least 11/19 had radiographic changes at baseline.^2^ Three of the 29 patients (10%) experienced an acceleration of rheumatoid nodulosis, which occurred simultaneously with improvement in their joint disease and was noted retrospectively.^2^ Thus, this was a small cohort from the early 1980s, with high disease burden and no access to modern disease-modifying therapies, limiting the generalisability of the reported MIAN incidence.

The second study is by Kerstens et al., a Dutch study published in 1992, in which 4 out of the 52 participating patients (8%) developed MIAN.^3^ This study shares the same limitations as the one by Kremer et al., as the patients had severe longstanding RA with failure to respond to the treatments available at the time.

The third study cited by the authors is the 2002 case series by Patatanian et al., including 27 patients.^4^ Notably, the authors themselves state that only 4 of the 27 cases were rated as “possible” based on the Naranjo scale, raising concerns about the strength of causality between methotrexate and the nodules.^4^ The Naranjo scale is a structured, question-based tool designed to assess the probability that an adverse drug reaction is truly attributable to the drug, as opposed to other confounding factors.

The estimated incidence of MIAN presented by Sabmaranian et al. appears to rely heavily on small case series conducted before the year 2000, often with uncertain causal attribution. Since then, numerous randomised controlled trials (RCTs) have been conducted in patients with established RA, and none have reported similarly high frequencies of MIAN.^5–8^ In these trials, methotrexate was used either as a comparator or as part of combination regimens with biologic DMARDs. The same applies to the recent TREAT EARLIER RCT, in which methotrexate was administered to patients with arthralgia at risk of developing RA.^9^ Additionally, in non-RA populations such as the large-scale CIRT trial, which included 2,391 patients treated with methotrexate versus 2,395 receiving placebo, no increased rates of MIAN were reported.^10^

The reported incidence of MIAN around 10%, as stated by Sabmaranian et al., seems inflated and based on outdated case series predating the biologic era, unsupported by higher-level evidence from modern RCTs. In my opinion, basing the estimation of the frequency of a clinical manifestation on case series or small cohorts carries a significant risk of selection bias, which may lead to an overestimation of its actual frequency.

Furthermore, reliable data are lacking for the size of the population at risk, making it difficult to accurately assess the true burden of the manifestation. Finally, confounding factors such as disease activity, risk of severe disease, and prior treatments are often not accounted for.

The use of methotrexate, whether as monotherapy or in combination with other synthetic or biologic DMARDs, has transformed the management of RA in recent years as part of the treat-to-target strategy and has significantly contributed to reducing the frequency of extra-articular manifestations.^11^ MIAN, by contrast, appears to be a rare occurrence, typically in RA patients on methotrexate monotherapy, with a frequency so low that it has not been captured in most RCTs in recent decades.
